# Size-Dependent Agonistic Interaction Patterns in Juvenile Male Swimming Crabs (*Portunus trituberculatus*)

**DOI:** 10.3390/ani16131958

**Published:** 2026-06-25

**Authors:** Nahayo Viateur, Litao Wan, Yuanyuan Fu, Hao Wang, Wenjun Xu, Jie He

**Affiliations:** 1 College of Fisheries, Zhejiang Ocean University, Zhoushan 316022, China; nahayoviateur04@gmail.com (N.V.); 17802520096@163.com (H.W.); 2 Zhejiang Marine Fisheries Research Institute, Zhoushan 316021, China; wan_066934@126.com (L.W.); wjxu1971@126.com (W.X.); 3 Ningbo Institute of Oceanography, Ningbo 315800, China; fuyuan0101@163.com

**Keywords:** agonistic behaviors, body size, *Portunus trituberculatus*, resource holding potential, size asymmetry, fighting intensity

## Abstract

Body size is a key determinant of agonistic interactions in swimming crabs. This study quantified agonistic interactions during pairwise contests among different size classes. Four size classes of male juveniles, *Portunus trituberculatus*, were examined (extra-large: 70.16 ± 1.12 g; large: 45.07 ± 1.15 g; medium: 25.30 ± 1.19 g; small: 15.08 ± 1.73 g; *n* = 12 per size class). The frequency and duration of agonistic behaviors and fighting intensity were recorded by a video recording system and analyzed. Larger crabs initiated more frequent and intense aggressive interactions, whereas smaller crabs exhibited mainly avoidance and submissive responses, particularly against larger opponents. The most intense and prolonged contests occurred between opponents with relatively small size differences. Conversely, highly size-mismatched pairs exhibited shorter, less intense interactions. These agonistic interactions were strongly size-dependent, consistent with Resource Holding Potential theory and size-contest dynamics. These behavioral patterns provide insights into social dynamics and inform aquaculture management practices.

## Simple Summary

An individual’s body size modulates aggressive interactions in social contexts. To examine the influence of body size on agonistic interactions, we investigated the behavior of male juvenile swimming crabs from size classes (extra-large, large, medium, and small) under controlled environmental conditions. The results showed that larger crabs initiated more aggressive behaviors and sustained longer, more intense interactions. However, smaller crabs often showed submissive behavior, especially when facing larger opponents. Fighting intensity and duration decreased as the size difference between opponents grew larger. The relative body size strongly shapes dominance hierarchies and contest strategies.

## 1. Introduction

Agonistic interactions include behaviors such as threat displays, physical contests, and submissive retreats. They are fundamental to social systems across animal taxa [1,2]. These behaviors contribute to dominance hierarchies [3], resource acquisition [4], mate competition [5], and shelter defense, highlighting their ecological importance, particularly in crustaceans. However, these interactions can impose costs, including reduced survival, growth, and physiological condition [6,7]. Several morphological factors influence agonistic behaviors. For instance, Su et al. [8] demonstrated that sex significantly affects aggression in swimming crabs, while Graham et al. [9] found that body size similarly influences aggression in virile crayfish *Faxonius virilis*. Physiological factors, including energetic status and neurochemicals such as dopamine and serotonin, further modulate the intensity of aggressive responses [10,11,12]. Phylogenetic and ecological context shape aggressiveness. For example, the Chilean species *Aegla abtao* and *A. denticulata* were generally more aggressive than their Brazilian counterparts *A. longirostri* and *A. manuinflata*. Notably, the morphologically distinct *A. denticulata* exhibited the lowest overall aggression [13]. 

Among these factors, body size is a critical determinant of contests and ecological interactions [14,15,16], as it correlates with Resource Holding Potential (RHP) and influences contest outcomes. RHP is defined as an individual’s ability to acquire and defend resources during competitive interactions [17,18]. Furthermore, it is commonly linked to traits such as body size, strength, and weapon morphology. RHP theory predicts that individuals with greater fighting abilities are more likely to initiate aggressive behaviors, escalate contests, and achieve dominance. In contrast, individuals with lower RHP are expected to adopt avoidance strategies [19]. Although larger males usually dominate, smaller individuals might win some contests, as larger males avoid the costs of escalated fighting [20]. Crustacean traits, such as chela size and carapace structure, further mediate fighting ability, reinforcing the role of size in shaping social hierarchy [21]. In mixed-species systems of Chinese mitten crabs (*Eriocheir sinensis*) and red swamp crayfish (*Procambarus clarkii*)*,* aggression increases with body size, whereas size differences influence fighting intensity [22]. Malavé et al. [23] revealed that male crayfish possess large chelae and exert greater pinching force than female conspecifics. Moreover, the ability to interlock chelae significantly affects the formation of the dominance hierarchy. For example, in *P. clarkii,* mismatched pairings demonstrated that smaller opponents with intact chelae defeat the larger crayfish with restricted chelae [24]. Size disparities affect the duration of agonistic encounters in crayfish *Pacifastacus leniusculus* [25]. Furthermore, agonistic encounters among male edible crabs (*Cancer pagurus)* revealed that winners exhibit dominance, while losers display submission [26].

*P. trituberculatus* is a common swimming crab widely distributed in the Bohai, Yellow, East China, and South China Seas [27]. Along China’s east coast, this species is widely cultured due to its commercial value. In 2022, its production in China reached 105,283 tons, representing a 4.35% increase from the previous year [28]. However, its aquaculture is constrained by aggressive behavior, which leads to frequent injury and cannibalism, thereby reducing survival and production yield [29]. Research on male swimming crabs suggests that aggression is life-stage dependent, with juveniles exhibiting higher levels than adults [30]. Previous studies have identified multiple factors influencing agonistic behavior. These include food availability [31], culture density and prior fighting experience [32], and territory size and dominance [33]. Swimming crabs grow through successive molting events. Asynchronous molting within a culture system often produces pronounced size variation among individuals [34,35]. Body size is closely associated with developmental stage. Consequently, individuals of different sizes coexist within culture systems, potentially increasing social interactions. However, the agonistic interactions among different size classes remain poorly understood. Size variation in crab culture systems can intensify resource competition and mortality, reducing production efficiency [36,37]. Based on predictions from RHP theory, we hypothesized that body size would influence agonistic interactions among juvenile male *P. trituberculatus*. Specifically, we predicted that larger individuals would exhibit more frequent, prolonged, and intense aggression, whereas smaller individuals would exhibit greater avoidance and submission towards larger opponents. We further predicted that contests between individuals with smaller size differences would escalate more than those involving large size asymmetries. To test these predictions, we quantified the frequency, duration, and intensity of agonistic interactions during pairwise encounters among different size classes under controlled conditions. Understanding how body size shapes interaction dynamics is essential for both behavioral ecology and aquaculture management.

## 2. Materials and Methods

### 2.1. Animal Collection and Maintenance

The experiment was conducted in August 2025 at the research base of the Zhejiang Marine Fisheries Research Institute. Juvenile male swimming crabs *P. trituberculatus* from four size groups were collected from outdoor ponds, where they hatched from captive broodstock and were reared under standard aquaculture conditions. Body weight was used as a proxy of overall body size, as it closely reflects growth and developmental status in juvenile swimming crabs. Moreover, body weight is routinely used to classify individuals in aquaculture, making it both biologically relevant and practically applicable. Juveniles were classified into four size classes: extra-large (70.16 ± 1.12 g, mean ± SD, *n* = 39), large (45.07 ± 1.51 g, *n* = 36), medium (25.30 ± 1.19 g, *n* = 38), and small (15.08 ± 1.73 g, *n* = 40). Each crab was housed individually in a cylindrical bucket (height = 32 cm; diameter = 20 cm) to prevent fighting before the experiment. Individuals exhibiting missing appendages or visible injuries were excluded prior to the experiment. Small holes were made in each bucket to facilitate water exchange, and crabs were acclimated for 5 days before the experiment. During the acclimation period, the buckets were submerged in a holding tank with the same dimensions as the experimental tank (height = 73 cm; diameter = 87 cm). Water depth was maintained at 45 cm, and oxygen was continuously supplied throughout the acclimation period. Fresh low-value fish was provided once daily (16:00) at 5% of body weight. The following morning at 07:00, uneaten feed was removed, and half of the tank water was replaced with clean seawater. Throughout the acclimation period, the water quality was maintained as follows: temperature 27.60 ± 0.8 °C, salinity 26.00 ± 0.5, pH 8.00 ± 0.3, dissolved oxygen > 5.00 mg/L, and photoperiod 12 L:12 D.

### 2.2. Experimental Setup and Observational Design

**Figure 1:**
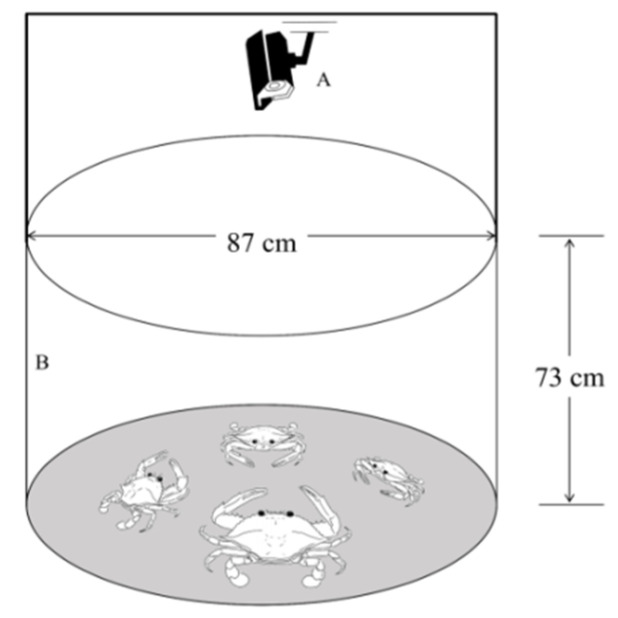
Agonistic behavior recording system (A: Camera and B: Experimental tank).

A video recording system was set up in the laboratory. The system comprised an infrared camera (Xiaomi hyperOS-BW500, 70mai Co., Ltd., Shenzhen, China; wavelength = 940 nm) and a cylindrical experimental tank (height = 73 cm; diameter = 87 cm). Before the experiment, only healthy male crabs in the intermolt stage were selected, weighed, and fasted for 24 h to standardize hunger levels and behavioral motivation before observations. Each crab was marked with a white dot on the carapace using acrylic paint to enhance contrast in video analysis, as individuals frequently changed position during interactions. Each experimental tank contained four crabs of different sizes: extra-large (70.02 ± 0.60 g; mean ± SD), large (45.03 ± 0.60 g), medium (25.04 ± 0.42 g), and small (15.08 ± 0.73 g). Twelve crabs from each size class (*n* = 12 per class) were used in the experiment. Crabs were randomly assigned to tanks to minimize systematic bias. Tanks were arranged in a 2 × 6 layout. Environmental conditions were standardized across all tanks to minimize positional effects. A camera was installed 60 cm directly above each observation tank to ensure complete visual coverage (Figure 1). Each tank (*n* = 12) was treated as an independent experimental unit and contained one crab from each size class. Each crab was used only once during the experiment and was assigned to a single tank, ensuring that no individual contributed data to more than one replicate. Because multiple interactions occurred among the same four individuals within a tank, observations from a given tank were not considered statistically independent. To avoid pseudoreplication, individual encounters were not treated as separate replicates. Instead, behavioral frequencies, durations, and tank-level means were used for subsequent statistical analyses. Thus, the 12 tanks represented 12 independent biological replicates. This design allowed simultaneous observation of pairwise interactions among size classes under standardized environmental conditions, reducing external variability and enabling the identification of size-related behavioral patterns. During the experiment, water quality parameters remained consistent with the acclimation period. However, aeration was reduced to minimize air bubbles and improve video clarity. Water depth was maintained at 40 cm, and the tank’s inner walls were painted blue to enhance the contrast in video analysis. A standardized 2 h evening window (20:00–22:00) was selected because portunids are nocturnal and most active during this period. Ambient noise was minimized to reduce disturbance. All interactions were recorded and stored for subsequent analysis.

### 2.3. Data Collection

Agonistic behaviors were quantified using a combination of manual recording and behavioral analysis software BORIS, version 9.5 [38]. Behavioral data were recorded for each focal crab during its interactions with opponent crabs in the same tank. Agonistic behaviors were scored only for direct pairwise interactions, and simultaneous interactions involving more than two individuals were excluded from analysis. Behaviors occurred sequentially during interactions, but each was defined by a distinct observable action and scored as a separate behavioral category. Two trained observers scored behaviors using standardized definitions. A subset of recordings was independently evaluated for inter-observer reliability, and any discrepancies were resolved before analysis. Agonistic behavior of swimming crabs was classified as either non-contact behaviors, including move to, move away, and cheliped display. Contact behaviors involved direct physical aggression (strike, grasp, push, and climb on), following Sneddon et al. [39] (Table 1). Fighting intensity was also scored for every encounter between opponents of different sizes. Fighting duration was measured from the initiation of aggressive behavior until both crabs ceased interacting. Fighting intensity was classified as strong, moderate, weak, or very weak [40] (Table 2).

**Table 1:** Description of agonistic behaviors shown by swimming crabs.

Agonistic Behavior	Description
Move to	One crab approaches another within interaction range without physical aggression (Figure 2a).
Cheliped display	One crab raises and extends its chelipeds toward the opponent, presenting open or closed chelae (Figure 2b).
Strike	One crab rapidly pinches the opponent with one or both chelipeds (Figure 2c).
Grasp	One crab grips the opponent’s carapace, chelipeds, or pereiopods with its chelae (Figure 2d).
Push	One crab applies sustained force using chelipeds or walking legs, resulting in displacement of the opponent (Figure 2e).
Climb on	One crab mounts the opponent by placing its body onto the opponent’s carapace (Figure 2f).
Move away	One crab increases distance from the other during or following an aggressive encounter (Figure 2g).
Non-contact behaviors	Consists of “ Move to”, “Move away”, and “Cheliped display”.
Contact behaviors	Consists of “ Strike”, “Grasp”, “Push”, and “Climb on”.

**Table 2:** Description of fighting intensity in the swimming crabs.

Intensity of Fighting	Description
Very weak	One crab approached another aggressively, and the other responded submissively without physical contact.
Weak	Both crabs showed aggression and made physical contact. The fight continued until the winner climbed on top of the other or the loser retreated.
Moderate	Both crabs exhibited aggression by pushing or grasping. The loser withdrew but continued to display threatening behavior, its cheliped still raised.
Strong	Both crabs showed aggression, pushing or grasping at each other. The loser repeatedly restarted the fight after retreating.

**Figure 2:**
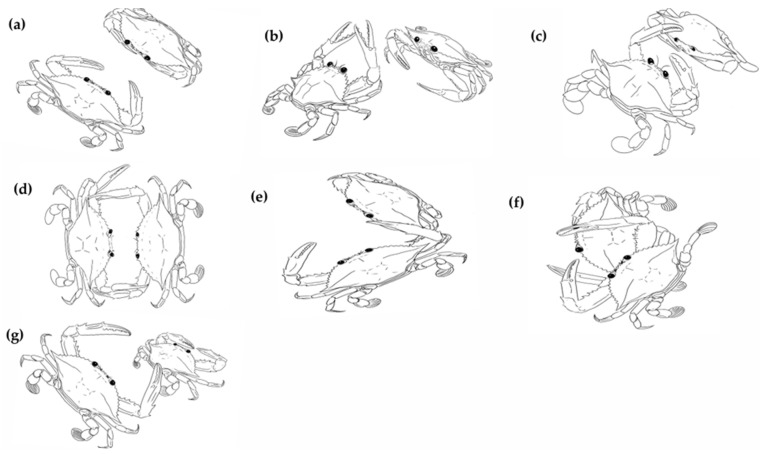
Diagram of agonistic behaviors in the swimming crab *P. trituberculatus*.

### 2.4. Data Analysis

Statistical analyses were performed in SPSS version 27.0 and GraphPad Prism version 9. Data are represented as mean ± SD. Normality and homogeneity were assessed using the Shapiro–Wilk test and Levene’s test, respectively. Behavioral frequency, duration, and fighting intensity were averaged per tank before statistical analysis to ensure independence among replicates. For each focal size class, separate two-way ANOVAs were conducted with opponent size (three levels corresponding to the three alternative size classes encountered) and behavior type (three levels for non-contact behaviors; four levels for contact behaviors) as fixed factors. Fighting intensity was analyzed using two-way ANOVA following aligned rank transformation (ART), with size combination and intensity level as fixed factors. Tukey’s post hoc tests were applied, and significance was set at *p* < 0.05.

## 3. Results

### 3.1. Body Size and Frequency of Non-Contact Behaviors

**Figure 3:**
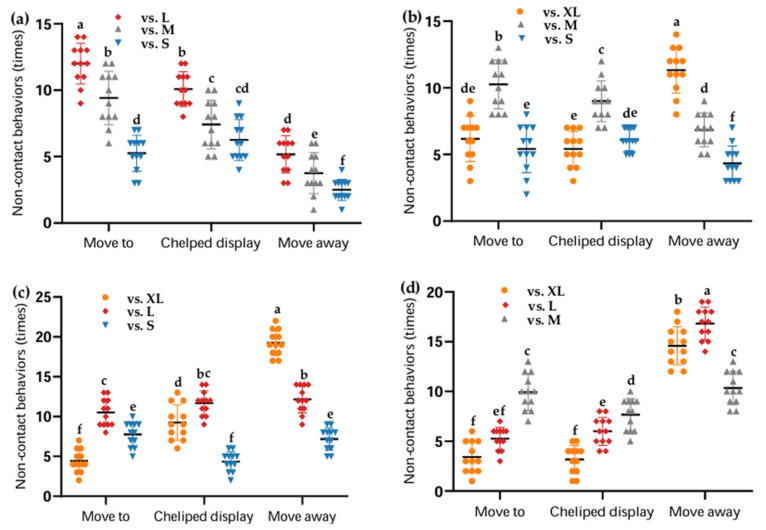
Frequency of non-contact behaviors among different class sizes: extra-large (**a**); large (**b**); medium (**c**); and small (**d**) male juveniles of *P. trituberculatus*. The bold central bar represents the mean, and the thin upper and lower bars represent the standard deviation. Different lowercase letters above indicate significant differences (*p* < 0.05). *n* = 12 tanks.

Two-way ANOVA showed significant interactions between behavior type and opponent size for all focal size classes (extra-large: *F* (4,11) = 6.80, *p* < 0.001, Figure 3a; large: *F* (4,11) = 38.98, *p* < 0.001, Figure 3b; medium: *F* (4,11) = 84.05, *p* < 0.001, Figure 3c; small: *F* (4,11) = 52.19, *p* < 0.001, Figure 3d). Overall, non-contact behaviors varied strongly with relative body size. Extra-large crabs showed higher frequencies of “move to” and aggressive display behaviors toward large opponents, whereas interactions with smaller opponents (medium and small) were less frequent. In contrast, “move away” responses in large, medium, and small crabs generally increased when facing larger opponents. Large and medium crabs displayed the strongest “move to” and “cheliped display” behaviors toward slightly smaller opponents, while “move away” behaviors were directed primarily toward larger crabs. Small crabs exhibited the clearest submissive patterns, “move away” most frequently during interactions with larger opponents, and increasing “cheliped display” as the size difference with their opponents shrank. Generally, “move to” and “cheliped display” behaviors were more common during interactions between individuals with a small size difference, whereas “move away” behaviors increased with a greater size asymmetry (*p* < 0.05; Figure 3).

### 3.2. Body Size and Frequency of Contact Behaviors

**Figure 4:**
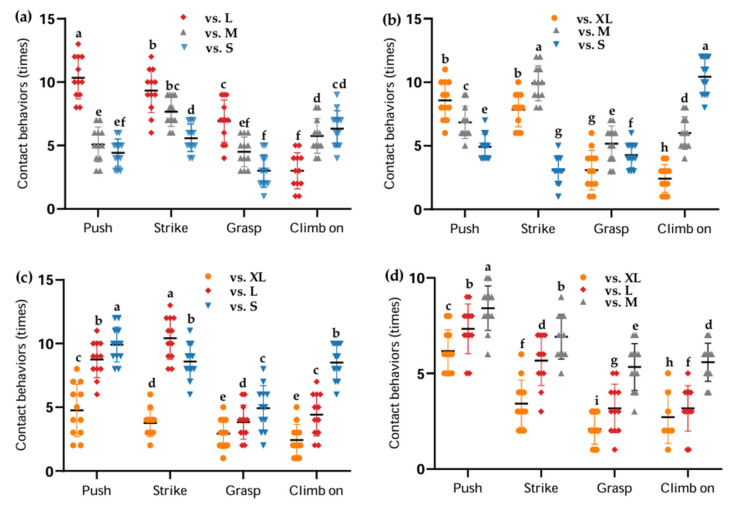
Frequency of contact behaviors among different class sizes: extra-large (**a**); large (**b**); medium (**c**); and small (**d**) male juveniles of *P. trituberculatus.* The bold central bar represents the mean, and the thin upper and lower bars represent the standard deviation. Different lowercase letters above indicate significant differences (*p* < 0.05). *n* = 12 tanks.

Two-way ANOVA identified significant interactions between behavior type and opponent size across all focal size classes (extra-large: *F* (6,11) = 27.01, *p* < 0.001, Figure 4a; large: *F* (6,11) = 117.16, *p* < 0.001, Figure 4b; medium: *F* (6,11) = 16.32, *p* < 0.001, Figure 4c; small: *F* (6,11) = 4.77, *p* < 0.001, Figure 4d). Contact behaviors were strongly modulated by relative body size. Extra-large crabs exhibited elevated frequencies of “strike”, “grasp”, and “push” behaviors during interactions with large individuals, whereas “climb on” behavior became more prevalent toward smaller opponents (medium and small). Large and medium crabs exhibited similar behavioral trends, with aggressive contact behaviors concentrated primarily in interactions with small opponents. In contrast, small crabs displayed limited contact aggression toward extra-large opponents but intensified physical interactions with medium-sized individuals. Collectively, contact aggression was most pronounced between closely size-matched crabs and declined progressively with increasing size asymmetry (*p* < 0.05; Figure 4).

### 3.3. Body Size and Duration of Non-Contact Behaviors

**Figure 5:**
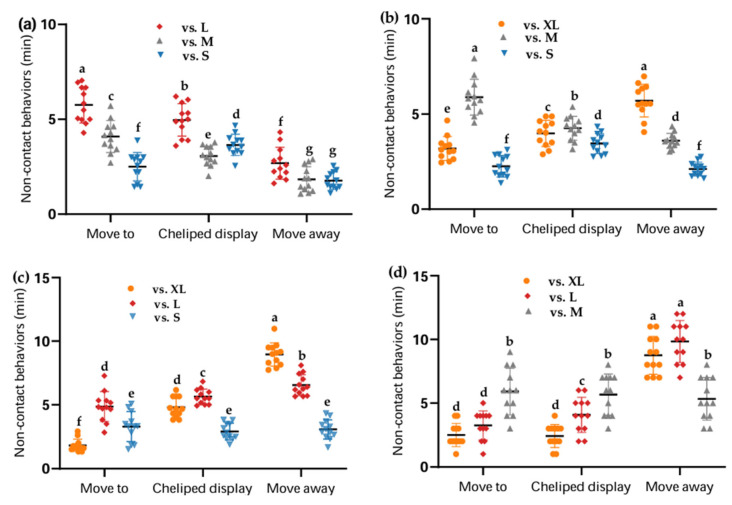
Duration time (min) of non-contact behaviors of different class sizes: extra-large (**a**); large (**b**); medium (**c**); and small (**d**) male juveniles of *P. trituberculatus*. The bold central bar represents the mean, and the thin upper and lower bars represent the standard deviation. Different lowercase letters above indicate significant differences (*p* < 0.05). *n* = 12 tanks.

Two-way ANOVA revealed significant interactions between behavior type and opponent size across all focal size classes (extra-large: *F* (4,11) = 11.54, *p* < 0.001, Figure 5a; large: *F* (4,11) = 51.23, *p* < 0.001, Figure 5b; medium: *F* (4,11) = 61.28, *p* < 0.001, Figure 5c; small: *F* (4,11) = 60.52, *p* < 0.001), Figure 5d). The duration of non-contact behaviors varied consistently with relative body size. Extra-large crabs maintained prolonged “move to” and “cheliped display” behaviors when interacting with large opponents, whereas interaction duration generally diminished toward smaller individuals (medium and small). Conversely, large, medium, and small crabs exhibited extended “move away” durations when facing extra-large opponents, indicating enhanced avoidance responses. Non-contact interactions between closely size-matched individuals were typically prolonged and characterized by assessment behaviors, whereas highly size-asymmetric encounters were shorter and dominated by retreat responses (*p* < 0.05; Figure 5).

### 3.4. Body Size and Duration of Contact Behaviors

**Figure 6:**
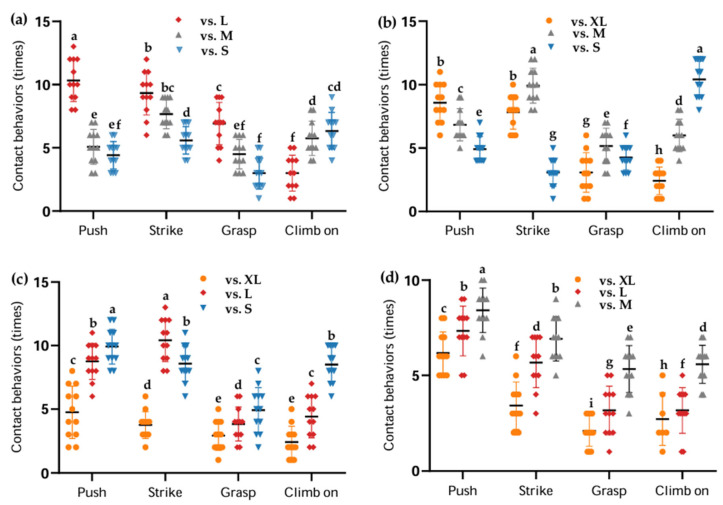
Duration time (min) of contact behaviors among different class sizes: extra-large (**a**); large (**b**); medium (**c**); and small (**d**) male juveniles of *P. trituberculatus.* The bold central bar represents the mean, and the thin upper and lower bars represent the standard deviation. Different lowercase letters above indicate significant differences (*p* < 0.05). *n* = 12 tanks.

Two-way ANOVA demonstrated significant interactions between behavior type and opponent size for all focal size classes (extra-large: *F* (6,11) = 31.68, *p* < 0.001, Figure 6a; large: *F* (6,11) = 47.98, *p* < 0.001, Figure 6b; medium: *F* (6,11) = 4.41, *p* < 0.05, Figure 6c; small: *F* (6,11) = 4.62, *p* < 0.05, Figure 6d). Contact behavior duration was strongly influenced by relative body size. Extra-large crabs engaged in longer “strike”, “push”, and “grasp” interactions with large opponents, whereas “climb on” duration increased markedly toward smaller individuals. Comparable tendencies were observed in large and medium crabs, which interacted predominantly with medium and small opponents, respectively. Small crabs displayed comparatively brief contact interactions with larger opponents, but sustained longer aggressive encounters with medium-sized individuals. In general, prolonged physical contests were more often between crabs with minimal size differences, whereas strongly size-asymmetric encounters were resolved more rapidly with lower escalation (*p* < 0.05; Figure 6).

### 3.5. Body Size and Fighting Intensity

**Figure 7:**
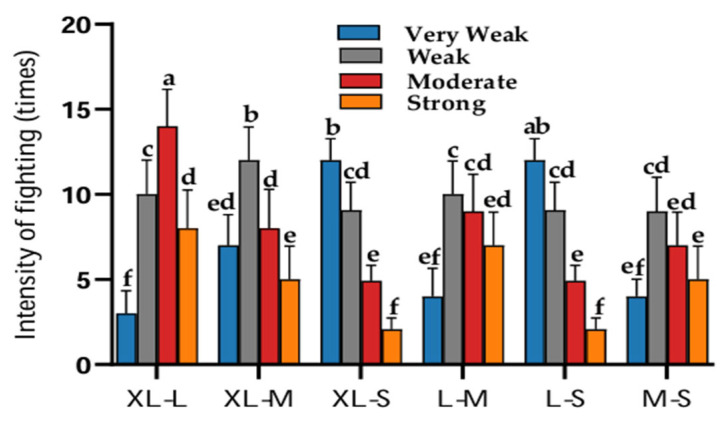
Intensity of fighting among different-sized pairs of juvenile male *P. trituberculatus*. Different lowercase letters above bars indicate significant differences (*p* < 0.05). *n* = 12 tanks. Note that XL-L stands for Extra-large versus Large, XL-M stands for Extra-large versus Medium, XL-S stands for Extra-large versus Small, L-M stands for Large versus Medium, L-S stands for Large versus Small, and M-S stands for Medium versus Small.

Two-way ANOVA following aligned rank transformation (ART) revealed a significant interaction between intensity level and size combination (*F* (12, 11) = 33.03, *p* < 0.001). “Very weak” intensity occurred most frequently in XL-S pairings. In contrast, “strong” and “moderate” intensities were more frequent in XL-L than in other pairings. “Weak” intensity was more frequent in the XL-M pairing than in other pairings (*p* < 0.05; Figure 7).

## 4. Discussion

Body size strongly influences agonistic interactions and contest outcomes in decapod crustaceans [41,42]. Juvenile male *P. trituberculatus* showed clear size-dependent behavioral patterns during agonistic interactions. Larger individuals showed greater persistence in aggression and initiated more interactions. They also engaged in longer, more intense contests, particularly against closer-sized opponents. Conversely, smaller crabs showed avoidance and submissive behaviors to larger rivals. These patterns support the Resource Holding Potential (RHP) theory, which links contest behaviors to relative fighting ability and body-size asymmetry, as reported in the hermit crab *Pagurus longicarpus* [43] and the mantis shrimp (stomatopoda) [44].

Our results corroborate previous studies showing that larger male crabs initiate aggressive interactions more frequently [45,46]. In the current study, larger crabs exhibited higher frequencies of “move to” and “cheliped display” behaviors, especially during interactions with medium-sized opponents. Similar patterns have been reported in other decapod species, where individuals with greater fighting capacity are more likely to approach and challenge rivals [47]. Threat display intensity also varied with the opponent’s relative size. Furthermore, comparable observations were reported in the Florida stone crab *Menippe mercenaria,* where larger individuals initiate aggressive and tactile display behaviors before escalation [48,49]. In addition, larger crabs occasionally submitted to and retreated from smaller opponents. According to Muramatsu and Koga [50], smaller decapods, such as the fiddler crab *Uca lactea*, can win contests against larger opponents by exerting greater effort and persistence. Small crabs predominantly showed “move away” behavior when interacting with larger opponents, but reduced this response toward medium-sized individuals. This pattern indicates that greater size variation triggers avoidance behavior. Similar shifts in dominance and submission across social contexts have been reported in the mud crab *Neohelice granulata* [51]. Increased retreat responses toward larger opponents have also been documented in other decapods, where highly size-asymmetric encounters are typically characterized by reduced escalation and shorter contests [52]. Medium crabs showed greater “move away” responses to larger opponents and more “move to” behavior toward smaller individuals. These findings indicate that agonistic responses varied with the opponent’s relative size, consistent with patterns reported in other decapods [53,54]. The repeated occurrence of approach behavior before escalation reflects an initial phase that precedes more intense aggression [55].

Size asymmetry strongly determines the transition from display behaviors to contact aggression [56]. Extra-large crabs exhibited more frequent “strike” and “push” behaviors toward large opponents, indicating greater escalation between more closer-sized individuals [57]. By contrast, small crabs reduced physical aggression toward larger opponents, implying that substantial size asymmetry suppresses confrontation in decapods. Similar patterns have been reported in other crustaceans, where smaller individuals avoid costly interactions with larger rivals, thereby contributing to size-based dominance hierarchies [54,58].

Contest duration also varied according to relative opponent size. Extra-large crabs spent more time in approach displays and contact behaviors when interacting with large opponents, suggesting more prolonged contests between closer-sized individuals [59]. In contrast, interactions involving small or medium crabs facing larger opponents were generally shorter and less escalated, showing that body size strongly influences contest persistence and aggressive investment [60].

Consistent with previous studies linking body size to fighting intensity in decapods [61], strong-intensity contests in this study occurred most frequently between extra-large and large individuals. This suggests that larger body size and enhanced weapon capacity promote escalation of contest intensity [62]. Interactions with pronounced size asymmetry were predominantly weak intensity, corroborating reports that unevenly matched opponents often resolve conflicts through displays or rapid withdrawal rather than prolonged physical combat [63,64]. The present findings support the predictions of RHP theory and demonstrate that body size is a key determinant of agonistic interactions in male juveniles of *P. trituberculatus*. Contest escalation and fighting intensity were greatest between closer-sized opponents, whereas smaller crabs predominantly avoided larger rivals. Given that size variation is common in aquaculture systems, size-dependent behavioral patterns may inform the development of management strategies to minimize aggression-related effects. Moreover, behavioral traits associated with aggression and dominance may provide useful indicators for selective breeding programs aimed at improving production performance in cultured swimming crabs.

## 5. Conclusions

Agonistic interactions in juvenile male *P. trituberculatus* are strongly influenced by body size and size asymmetry. Larger individuals showed greater aggressive investment and dominance behavior, whereas smaller crabs primarily adopted avoidance strategies. These behavioral patterns have important implications for both behavioral ecology and aquaculture management. In aquaculture systems, variation in body size can influence the frequency, duration, and intensity of agonistic interactions among individuals. Therefore, consideration of size structure may help improve the management of social interactions and reduce aggression-related losses, such as injuries and cannibalism, in swimming crab culture. Future studies should investigate the effects of size symmetry and asymmetry in both sexes and conduct long-term research on how body size affects agonistic interactions among swimming crabs.

## Data Availability

The data presented in this study are available in the article. Raw data can be obtained from the corresponding author upon reasonable request.
